# Type II diabetes and impaired glucose tolerance due to severe hyperinsulinism in patients with 1p36 deletion syndrome and a Prader-Willi-like phenotype

**DOI:** 10.1186/1471-2350-15-16

**Published:** 2014-01-30

**Authors:** Stefano Stagi, Elisabetta Lapi, Marilena Pantaleo, Francesco Chiarelli, Salvatore Seminara, Maurizio de Martino

**Affiliations:** 1 Health’s Sciences Department, University of Florence, Anna Meyer Children’s University Hospital, Florence, Italy; 2 Genetics and Molecular Medicine Unit, Anna Meyer Children’s University Hospital, Florence, Italy; 3 Department of Paediatrics, University of Chieti, Chieti, Italy; 4 Paediatric Endocrinology Unit, Anna Meyer Children’s University Hospital, viale Pieraccini 24, Florence 50139, Italy

**Keywords:** Monosomy 1p36, Deletion 1p36, Developmental delay, Mental retardation, Seizures, Obesity, Hyperinsulinism, Impaired glucose tolerance, Hyperphagia, Prader-Willi-like phenotype

## Abstract

**Background:**

Deletion of the subtelomeric region of 1p36 is one of the most common subtelomeric deletion syndromes. In monosomy 1p36, the presence of obesity is poorly defined, and glucose metabolism deficiency is rarely reported. However, the presence of a typical Prader-Willi-like phenotype in patients with monosomy 1p36 is controversial.

**Case presentation:**

In this report, we describe two female patients, one who is 6 years 2 months of age and another who is 10 years 1 month of age, both referred to our hospital for obesity and a Prader-Willi-like phenotype. These patients presented with severe obesity (body mass index [BMI] was 26.4 and 27.7, respectively), hyperphagia and developmental delay. Analysis of basal hormone levels showed normal thyroid function and adrenal function but considerable basal hyperinsulinism (the insulin levels were 54.5 and 49.2 μU/ml, respectively). In patient 1, glycaemia was 75 mg/dl (HOMA-R 10.09), and the HbA1c level was 6.1%; in patient 2, glycaemia was 122 mg/dl, and the HbA1c level was 6.6% (HOMA-R 14.82). An oral glucose tolerance test demonstrated impaired glucose tolerance and diabetes mellitus with marked insulin resistance (the peak insulin level for each patient was 197 and 279 μU/mL, respectively, while the 120’ insulin level of each patient was 167 and 234 μU/mL, respectively).

**Conclusion:**

some patients with monosomy 1p36 may show Prader-Willi-like physical and physiologic characteristics such as obesity and hyperinsulinism with impaired glucose metabolism, which can cause type II diabetes mellitus. Further studies are necessary to evaluate these findings.

## Background

Deletion of the subtelomeric region of 1p36 is one of the most common subtelomeric deletion syndromes, with an estimated incidence of 1 in 5000 to 1 in 10,000 live births [1-3]. The four classes of rearrangements identified in individuals with monosomy 1p36 in order of frequency are as follows: apparently pure/simple terminal deletions (67%), derivative chromosomes (16%), interstitial deletions (10%), and complex rearrangements (7%) [4].

Monosomy 1p36 is responsible for a cluster of characteristic clinical features including mental retardation and multiple congenital abnormalities caused by haploinsufficiency of numerous contiguous genes [1], such as deep-set eyes, straight eyebrows positioned low on the supra-orbital ridges, asymmetric ears, a pointed chin, and a flat nasal bridge [1]. Additional features include cardiomyopathy, hearing impairment, seizures [5,6], and a variety of neoplasms due to the loss of numerous tumour-suppressor genes that are located in this chromosomal region [7,8].

In monosomy 1p36, the occurrence of both obesity and or glucose metabolism disorders is rarely reported. However, the presence of a typical Prader-Willi-like phenotype in patients with 1p36 monosomy is controversial.

## Case presentation

In this report, we describe two female subjects with monosomy 1p36 and Prader-Willi-like phenotype who also exhibit type II diabetes and impaired glucose tolerance due to hyperinsulinism. We provide data both for and against this possible association. Written informed consent was obtained from the parents before publication of this case report and any accompanying images.

### Clinical report 1

A female patient, 6 years 2 months of age, was referred to our Paediatric Endocrinology Unit for severe obesity.

The proposita was the third-born child (the first pregnancy was interrupted because of a disorder involving chromosome 7) of a nonconsanguineous marriage who was delivered normally at 40 weeks after an uncomplicated pregnancy. The family history was unremarkable, particularly for obesity, which was present only in a paternal uncle. The Apgar score was 5^I^-7^V^-8^X^, and the infant required oral and gastric suctioning, as well as oxygen and stimulation upon delivery to treat cyanosis. Birth weight was 2.175 kg (-2.67 SDS; <3^rd^ centile), length was 44 cm (-2.92 SDS; <3^rd^ centile), and head circumference was 32 cm (-2.01 SDS; <3^rd^ centile). The neonatal screening for congenital hypothyroidism was negative.

She presented with abnormal psychomotor development, as she sat upright at 8 months, began to walk independently at 15 months and began to use language at 15 months.

At 6 yrs, 2 months of age, this patient weighed 32.000 kg (1.73 SDS; > > 97^th^ centile), her height was 110 cm (-1.34 SDS; 3-10^th^ centile), and her occipital-frontal circumference was 45.8 cm (-2.65 SDS; <3^rd^ centile). This patient’s BMI was 26.4 (2.41 SDS; > > 97^th^ centile), and the pubertal staging was defined as B1-PH2-3-AH2. Her blood pressure evaluation showed both systolic and diastolic hypertension (124/85 mmHg).

**Figure 1:**
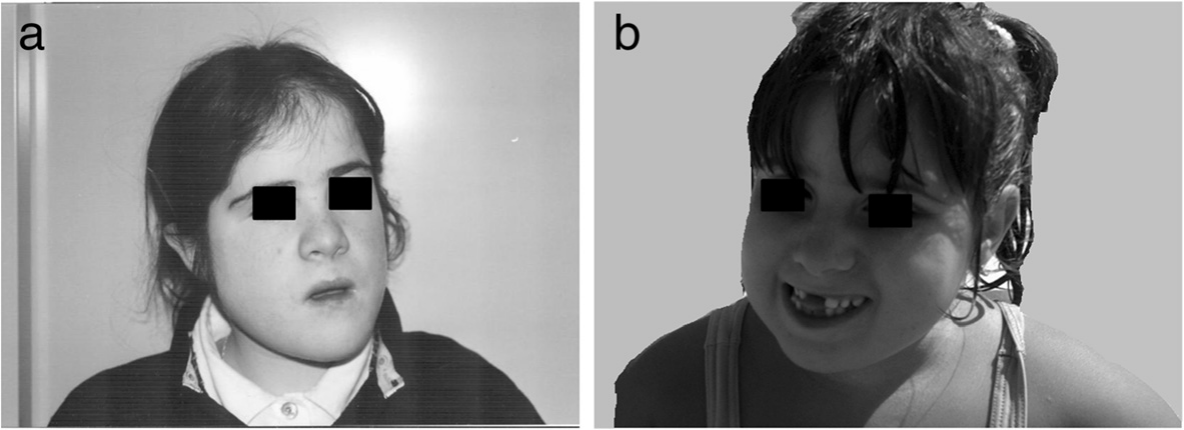
**Case 1 at 4 years 2 months of age (1a), and case 2 at 6 years 8 months of age (1b).** In these patients, there is evidence of some of the typical facial dysmorphisms of 1p36 deletion syndrome with Prader-Willi-like phenotype.

Numerous dysmorphic features were observed, including a small mouth with heaped-up palate, a small chin, a small folded ear, straight eyebrows, fifth finger clinodactyly and short toes. Examination of the subject's mother revealed some mild dysmorphism, clinodactyly of the fifth finger and a mild learning disability (Figure 1a; Table 1).

**Table 1:** Clinical findings in patients with 1p36 deletion syndrome and Prader-Willi-like phenotype

**Clinical findings**	**Case 1**	**Case 2**	**D’Angelo *et al.*, 2006 [**[11]**]**	**D’Angelo *et al.*, 2010 [**[23]**]**	**Eugster *et al.*, 1997 [**[21]**]**	**Tsuyusaki *et al.*, 2010 [**[24]**]**	**Tsuyusaki *et al.*, 2010 [**[24]**]**
Microcephaly	+	+	-	-	-	-	-
Brachycephaly	+	+	-	-	+	-	-
Straight eyebrows	+	+	+	+		+	+
Deep-set eyes	+	+	+/-	+/-	+	+	+
Epicanthal folds	+	+	-	-	-	-	-
Broad nasal root/bridge	+	+	-	-	+	+	-
Posteriorly rotated/low-set/abnormal ears	+	+	-	-	-	+	-
Pointed chin	+	+	-	-	+	+	-
Midface hypoplasia	+	+	-	-	+	+	+
Short feet	+	+	+	+	+	+	+
Developmental delay	+	+	+	+	+	+	+
Mental retardation	+	+	+	+	+	+	+
Hypotonia	+	+	+	+	+	+	+
Seizures	+	+	-	-	+	+	+
Brain abnormalities	-	-			-	+	+
Sensorineural deafness	+/-	+/-	-	-	+	+/-	-
Strabismus	+	+	-	-	+	-	-
Expressive language (poor/absent)	+	+	+	+	+	+	+
Behavioural disorders	+	+	+	+	+	+	+
Heart abnormalities	-	-	-	-	ND	+	-
Renal abnormalities	-	-	-	-	ND	-	-
External genitalia abnormalities	-	-	-	-	ND	-	-
Poor neonatal weight	+	+	+	+	+/-	+	-
Poor neonatal length	+	+	+	+	ND	-	+
Hyperphagia	+	+	+	+	+	+	+
Obesity	+	+	+	+	+	+	+
Hyperinsulinism	+	+	ND	ND	ND	ND	ND
Thyroid disorders	-	-	+ (thyroid nodules)	+	ND	ND	ND

+ = present.

- = not present.

ND = not determined.

Assessment of basal hormone levels showed normal free thyroxine (1.63 ng/dL; range 0.8-1.9), TSH (3.51 μIU/mL; range, 0.4-4.0), cortisol (12.3 μg/dL; range 5-19) and ACTH (21.1 ng/L; range 9.0-52.0) values. The blood count was also normal. Her blood chemistry was AST: 45 IU/L, ALT: 35 IU/L, γ-GTP: 27 IU/L, HbA1c: 6.1%, TG: 132 mg/dL, total cholesterol: 195 mg/dL, HDL: 51 mg/dL, insulin: 54.5 μU/ml, glycaemia: 102 mg/dl (HOMA 13.72). A screening for coeliac disease was negative (IgA 142 mg/dl; tTG 0.2 U/mL), but an oral glucose tolerance test indicated hyperinsulinism (peak insulin level was 297 μU/mL), impaired glucose tolerance (120’ glycaemia 179 mg/dl), and marked insulin resistance (120’ insulin level 193 μU/mL).

Routine cytogenetic investigations revealed an apparently normal female karyotype (46, XX). Chromosomal studies, also using FISH, ruled out a 15q11.2-q13 deletion. Tests for methylation and the presence of maternal uniparental disomy were both negative.

Given the numerous clinical features observed in this subject, a FISH analysis utilising 41 subtelomere probes was performed (Genzyme laboratories (Hawthorne, NY)) using Vysis probes (Downers Grove, IL). The results were confirmed with a chromosome 1p subtelomere probe (1pSUBTEL; Vysis) and the D1Z2 midi-satellite probe with repeats in band 1p36 (Oncor), which is used to confirm a common deletion interval (1p36.3).

**Figure 2:**
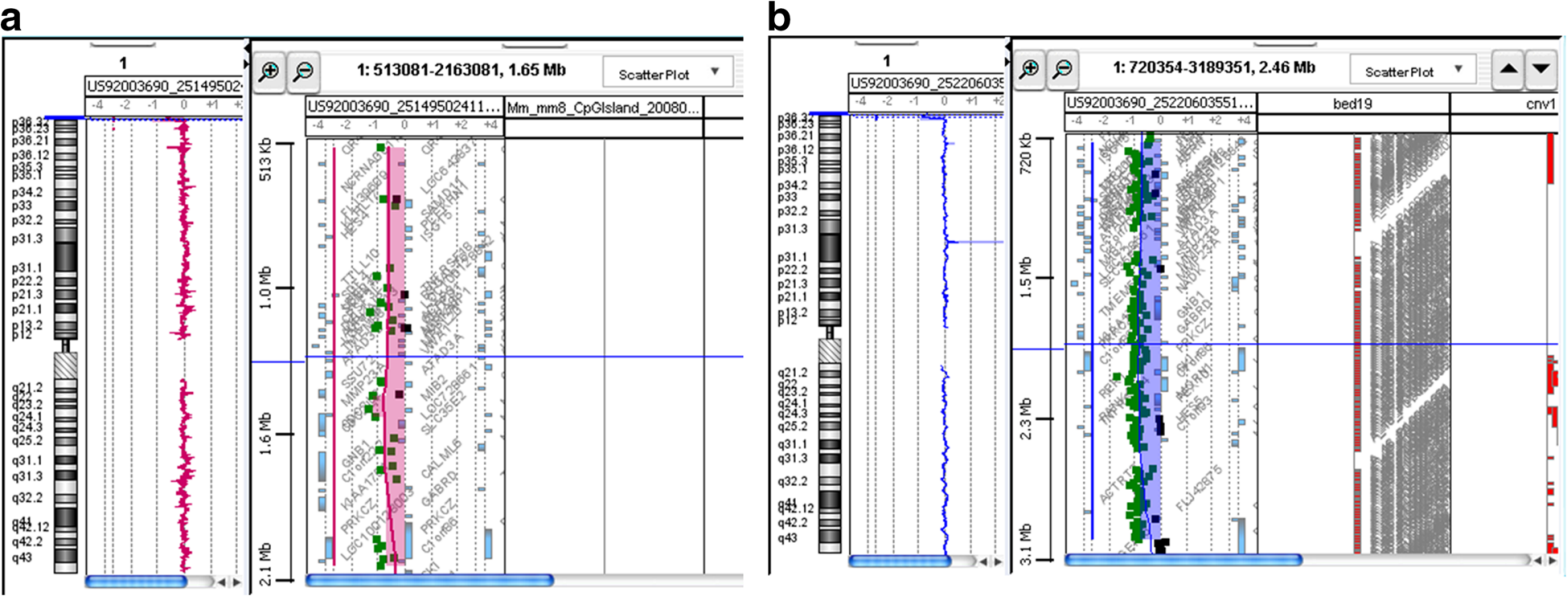
Array-CGH analysis showing a deletion of approximately 1.5 Mb that involved the 1p36.33 region, with the breakpoint falling between 554,268 bp and 2,133,973 bp (2a), and a deletion of approximately 2.5 Mb that involved the 1p36.33 – 1p36.32 region, with the breakpoint falling between 564,424 bp and 3,111,905 bp (2b).

Molecular karyotyping was performed by an array-CGH analysis using the proband's DNA and an Agilent 44 K array platform with a resolution of approximately 100 kb. Based on the physical mapping positions designated in the March 2006 assembly (NCBI36/hg18) of the UCSC Genome Browser, this analysis showed a deletion of approximately 1.5 Mb that involved the 1p36.33 region, with the breakpoint falling between 554,268 bp (first-deleted oligomer) and 2,133,973 bp (last-deleted oligomer) (Figure 2a).

### Clinical report 2

The female patient, 10 years 1 month of age, was referred to the Genetics and Molecular Medicine Unit of Anna Meyer Children’s University Hospital for severe obesity and suspected Prader-Willi syndrome.

The proposita was the second-born child of a nonconsanguineous marriage who was delivered normally at 39 weeks after an uncomplicated pregnancy. The family history was unremarkable in terms of obesity. The patient has a brother with normal staturo-ponderal growth who was 6 yrs old at the time of this study.

The Apgar score was 6^I^-9^V^-9^X^; as an infant, she required oral suctioning, oxygen and stimulation at delivery for treatment of cyanosis. Her birth weight was 2.360 kg (-1.92 SDS; <3^rd^ centile), her length was 44.6 cm (-2.24 SDS; <3^rd^ centile), and her head circumference was 32 cm (-1.90 SDS; <3^rd^ centile). Central hypotonia occurred soon after birth. The screening for neonatal congenital hypothyroidism was negative, but she experienced recurrent respiratory infections throughout the first year of life.

At 6 months of age, persistence of patent ductus arteriosus (PDA), which lead to pulmonary hypertension, was diagnosed; PDA was closed at 7 months. She presented with abnormal psychomotor development, as she began to sit upright at 9 months, walk independently at 17 months and demonstrated use of language at 19 months. Bilateral neurosensory deafness was diagnosed at 1 year of age. The child developed significant hyperphagia.

At 10 yrs 1 month of age, the proposita showed a phenotype consistent with Prader-Willi syndrome: the patient weighed 52.600 kg (1.65 SDS; 90-97^th^ centile), her length was 131.8 cm (-1.18 SDS; 10^th^ centile), and her BMI was 27.7 (2.02 SDS; > > 97^th^ centile). The pubertal staging was defined as B1-PH2-3-AH2. Blood pressure evaluation showed systolic and diastolic hypertension (124/85 mmHg).

At the evaluation, she was observed to have numerous dysmorphic features, including substantial truncular obesity, scoliosis, small hands and feet, a small mouth with heaped-up palate, a small chin and a small folded ear, straight eyebrows, fifth finger clinodactyly and short toes, and a mild learning disability. Examination of the mother revealed some mild dysmorphism, clinodactyly of the fifth finger, and a mild learning disability (Figure 1b).

An analysis of basal hormone levels showed normal free thyroxine (13.7 pmol/L; range 10.3-19.4 pmol/L), TSH (2.6 mIU/L; range, 0.4-4.0), cortisol (14.1 ug/dL; range 5.0-19.0) and ACTH 14.1 (range 9.0-52.0 ng/L) values. The blood count was also normal, and her blood chemistry was as follows: AST: 20 IU/L, ALT: 21 IU/L, γ-GTP: 32 IU/L, HbA1c: 5.5%, TG: 146 mg/dL, total cholesterol: 259 mg/dL, HDL: 45 mg/dL, prolactin: 271 mIU/L, HbA1c: 6.6%, insulin: 49.2 μU/ml, glycemia 122 mg/dl (HOMA-R 14.82). The screening for coeliac disease was negative (IgA 165 mg/dl; tTG 0.4 U/mL), and an oral glucose tolerance test indicated diabetes mellitus (120’ glycaemia 234 mg/dl), with marked hyperinsulinism and insulin resistance (peak insulin level was 279 μU/mL, 120’ insulin level 167 μU/mL).

Routine cytogenetic investigations revealed an apparently normal female karyotype (46, XX). Chromosomal studies, which also used FISH, ruled out a 15q11.2-q13 deletion found in Prader-Willi syndrome. The hypophyseal RMN revealed an empty sella.

At 12 yrs, 8 months, her height was 141 cm (-2.10 SDS; < 3^rd^ centile), BMI was 29.9 (2.18 SDS; > 97^th^ centile), and pubertal staging was described as B2-3-PH4-AH2. Menarche started at 13 years of age.

At 16 years of age, her height was 145 cm (-2.82 SDS; < 3^rd^ centile), her weight was 56.000 kg (0.05 SDS; 50-75^th^ centile), and pubertal staging was found to be B5-PH5-AH3.

Tests for methylation and the presence of maternal uniparental disomy were both negative. Therefore, given the numerous clinical features observed in this subject, a FISH analysis utilising 41 subtelomere probes was performed (Genzyme laboratories (Hawthorne, NY), using Vysis probes (Downers Grove, IL). The results were confirmed with a chromosome 1p subtelomere probe (1pSUBTEL; Vysis) and the D1Z2 midi-satellite probe with repeats in band 1p36 (Oncor). The D1Z2 probe is used for the confirmation of a common deletion interval (1p36.3). Array-CGH was performed using the Agilent Human Genome CGH microarray kit 180 K, with a resolution of approximately 40 kb. Break point positions were reported according to Hg19, with build 37 falling between 564,424 bp (first-deleted oligomer) and 3,111,905 bp (last-deleted oligomer), which showed a deletion of approximately 2.5 Mb that involved the 1p36.33 – 1p36.32 region (Figure 2b).

## Conclusion

Monosomy 1p36 is a common deletion syndrome that produces specific physical characteristics, such as distinctive facial anomalies (pointed chin, flat nose, low-set ears), cardiovascular malformations (atrial septal defect, patent ductus arteriosus, tetralogy of Fallot), central nervous system defects (mental retardation, cranial nerve abnormalities, sensorineural hearing loss), neonatal hypotonia, cortical dysplasia, and seizures [2,9,10]. Affected individuals can display outbursts, a tendency towards violent and self-injurious behaviour, as well as autistic-like behaviours [5].

In patients with monosomy 1p36, the presence of obesity has been poorly defined, and glucose metabolism disorders have been infrequently reported to date. For example, D'Angelo *et al.*[11] identified 1 case of diabetes in a 13-year old patient, from within a group of 41 patients with a Prader-Willi-like phenotype, who had a pure 1p36 deletion. However, a study that evaluated the glucose metabolism in subjects with 1p36 deletion syndrome had not been performed until our current study.

Our case reports confirm this association and suggest that this phenotype may be associated with obesity in some 1p36 patients. We also present evidence that hyperinsulinism and impaired glucose tolerance or diabetes mellitus may be present in these patients and that these features can occur in younger patients.

In fact, genome-wide linkage studies suggest that the 1p36 region shows a strong correlation with several traits normally associated with metabolic syndrome (MetS) [12], such as hyperlipidaemia, diabetes, obesity, arterial hypertension, and BMI cluster [13,14].

Prader-Willi-like phenotype has been described in patients with chromosomal duplications [15,16], Fragile X syndrome [17], udp(14)mat [18,19], and 6q deletion syndrome [20].

Obesity and/or hyperphagia are features rarely described in patients with 1p 36 deletion [1,11,21-24], but correlations between the extents of the deleted chromosomal segments have not been comprehensively provided. A hypothesis for the similarity between patients with a 1p36 deletion and those with Prader-Willi syndrome, as well as for the existence of two different phenotypes for a 1p36 microdeletion, has been suggested [23]. Contrary to its manifestation in 1p36 deletion syndrome, obesity is a common clinical sign associated with mental retardation, and is clearly a public health problem. Thus, a suitable question to ask is whether these patients, who were described as being obese, are really representative of this particular deletion syndrome [25].

To help paediatric neurologists and other professionals in the recognition of this emerging and common chromosomal syndrome and to identify the possible overlap of clinical presentations between patients with 1p36 deletion syndrome and patients with a Prader-Willi like phenotype, we have reviewed published articles that describe patients with these features.

D’angelo *et al*. [23] described samples from 26 patients with Prader-Willi-like phenotype that were analysed with a 1p-specific subtelomeric probe, where one terminal deletion was identified in one patient. This patient did not have any of the obvious features that resembled this monosomy, but her clinical and behavioural features were quite similar to those observed in patients with PWS, except for the presence of normal sucking at birth. The extent of the deletion could be limited to the most-terminal 2.5 Mb of 1p36, within the chromosomal region 1p36.33–1p36.32, that is smaller than that usually observed in monosomy 1p36 patients. Slavotinek *et al.*[5] reviewed 39 cases reported to have pure 1p36 deletions and 2 cases (5.1%) that demonstrated the Prader-Willi-like phenotype. Shapira *et al.*[1] described 13 cases of the pure 1p36 deletion and found 2 cases (15%) of obesity. Therefore, chromosome 1p36.33 deletion should be investigated in patients with hypotonia, developmental delay, obesity and/or hyperphagia and behavioural problems who test negative for PWS, which might suggest non-classical manifestations of this disorder in these patients [23].

Thus far, no individual gene within this region has been conclusively determined to be causative of any component of the phenotype [23]. However, in patients with 1p36 deletion syndrome and PWS-like phenotype, the possible involvement of the terminal 4 Mb of chromosome 1p36 may be postulated [24].

Interestingly, D’Angelo *et al.*[23] have found that some genes located near 1p36 may be involved in carbohydrate or lipid metabolism or insulin signalling. Some of these genes may be implicated in the Prader-Willi-like phenotype typical of some monosomy 1p36 patients through a mechanism of either haploinsufficiency, position effect or gene interactions (epistasis) [23]. This may be important because the 1p34 –36 region has shown significant genome-wide linkage to premature myocardial infarction [26].

Approximately 90% of the 1p rearrangements are less than 10 Mb in size [3], and it is assumed that quite a number of subjects are misdiagnosed [3]. Moreover, several subjects diagnosed with monosomy 1p36 have been reported to have mild or atypical phenotypes or presenting features that overlap with other genetic syndromes [11,27-29].

In conclusion, our case reports confirm that some patients with 1p36 monosomy may show a Prader-Willi-like physical phenotype and that this aspect may therefore be overlooked, which may result in this condition being underdiagnosed. These patients may show such characteristics as obesity with hyperinsulinism and impaired glucose metabolism. Further studies are necessary to further evaluate this aspect.

### Consent statement

Written informed consent was obtained from the parents of the patients for publication of this case report and any accompanying images.

## Competing interests

The authors declare that they have no competing interests.

## Authors’ contributions

SStagi carried out the endocrinological evaluation, conceived of the study and participated in its design. EL performed the clinical genetic diagnosis. SSeminara performed the endocrinological evaluation. MP performed the molecular genetic studies. FC participated in the endocrinological evaluation. MdM participated in the endocrinological evaluation and participated in its coordination. All authors read and approved the final manuscript.

## Pre-publication history

The pre-publication history for this paper can be accessed here:

http://www.biomedcentral.com/1471-2350/15/16/prepub
